# The curative effects of shortwave diathermy on treating Novel coronavirus (COVID-19) pneumonia: A structured summary of a study protocol for a randomised controlled trial

**DOI:** 10.1186/s13063-020-04534-5

**Published:** 2020-07-03

**Authors:** Mohammad Nasb, Zulfiqar Ali Sayed Shah, Liangjiang Huang, Qian Li, Hong Chen

**Affiliations:** 1 Department of Rehabilitation Medicine, Tongji Hospital, Tongji Medical College, Huazhong University of Science and Technology, Wuhan, 430030 People’s Republic of China; 2 Department of Physical Therapy, Health Science Faculty, Albaath University, Homs, Syria; 3 WHO Collaborating Centre for Training and Research in Rehabilitation, Tongji Hospital, Tongji Medical College, Huazhong University of Science and Technology, Wuhan, 430030 People’s Republic of China

**Keywords:** COVID-19, Randomised controlled trial, Protocol, Coronavirus, Ultra short-wave diathermy, Pneumonia

## Abstract

**Objectives:**

To evaluate the therapeutic effects of ultra-short-wave diathermy (SWD) on COVID-19 pneumonia. The hypothesis is that SWD may minimise pneumonic inflammation and shorten the duration of the time to positive-to-negative conversion of COVID-19 nucleic acid test.

**Trial design:**

This is a single centre, 2-arm (1:1 ratio), evaluator blinded, parallel group design superiority randomised, controlled clinical trial.

**Participants:**

The inclusion criteria were: (1) Age 18-65 years, (2) COVID-19 nucleic acid test is positive, (3) Lung CT showed multiple patchy ground glass shadows or other typical manifestations of both lungs. The exclusion criteria were: (1) Patients who need ICU management, (2) Positive tests for other pathogens such as Tuberculosis, Mycoplasma, (3) Patients with respiratory failure or requiring mechanical ventilation, (4) Patients with metal implants or pacemakers, (5) Those with shock (6) Those that have bleeding tendency or active bleeding in the lungs, (7) Patients with multiple organ failure who need ICU monitoring and treatment, (8) Cancer patients and those with severe underlying diseases, (9) Pregnant or lactating women, (10) Patients with severe cognitive impairment who cannot follow the instructions to complete the treatment, (11) Those without signed informed consent and (12) Those with other contraindications to short wave. This study will be conducted in Tongji Hospital, Caidian, Wuhan, People’s Republic of China.

**Intervention and comparator:**

The experimental group will be given the nationally recommended standard medical treatment + ultra-short-wave diathermy treatment. Ultra-short-wave therapy treatment will be performed through application of ultra-short-wave therapy machine electrodes on the anterior and posterior parts of the trunk for 10 minutes, twice a day for 12 consecutive days. The comparator will be the control, not receiving ultra-short-wave therapy, and will be given only the nationally recommended standard medical treatment.

**Main outcomes:**

The primary outcome measures will be time to positive-to-negative conversion of COVID-19 nucleic acid test by pharyngeal swab, in days assessed at 7^th^, 14^th^ ,21^st^ and 28^th^ days. The secondary outcome measures include nucleic acid test rate and recovery from symptoms, Vital signs assessment, Computed Tomography, Complete blood count, serum analysis and SIRS scale scores. Blinded evaluation will be at baseline (the day of starting ultra-short-wave diathermy) and after 28 days following the interventions.

**Randomisation:**

A Randomization plan will be generated online on www.randomization.com using permuted blocks method, by a statistician who will not be part of the study. Small blocks of various sizes will be used. Patients will be randomized (1:1) between the experimental and control groups

**Blinding (masking):**

This will be an evaluator blinded study. Due to the nature of the intervention, blinding of patients and healthcare workers is not possible.

**Numbers to be randomised (sample size):**

A total of 410 patients will be randomised in 1:1 ratio to two groups: experimental group (n=205) and control group (n=205).

**Trial Status:**

Protocol version 1 was approved on 02/12/2020. Recruitment for this trial began on 02/18/2020 and will be ongoing till the required sample size is reached. The analysis deadline is August 2020.

**Trial registration:**

This randomised controlled trial has been prospectively registered with the Chinese Clinical Trials (ChiCTR2000029972) on 17 February 2020.

**Full protocol:**

The full protocol is attached as an additional file, accessible from the Trials website (Additional file 1). In the interest in expediting dissemination of this material, the familiar formatting has been eliminated; this Letter serves as a summary of the key elements of the full protocol.”

The study protocol has been reported in accordance with the Standard Protocol Items: Recommendations for Clinical Interventional Trials (SPIRIT) guidelines (Additional file 2).

## Supplementary information

**Additional file 1.** Full Study Protocol.

**Additional file 2.** SPIRIT 2013 Checklist: Recommended items to address in a clinical trial protocol and related documents.

## Data Availability

The data set will be made available upon reasonable request to the corresponding author after completion of the study.

