# Supplementary material for: The curative effects of shortwave diathermy on treating Novel coronavirus (COVID-19) pneumonia: A structured summary of a study protocol for a randomised controlled trial
Source: Trials. 2020 Jul 3;21:609. doi: 10.1186/s13063-020-04534-5 (PMC7332534; doi:10.1186/s13063-020-04534-5)
Supplement: Supplementary file 1 — Additional file 1. Full Study Protocol. [file 13063_2020_4534_MOESM1_ESM.docx]

**The curative effects of shortwave diathermy on treating Novel coronavirus (COVID-19) pneumonia: Study protocol for a randomised controlled trial**

Mohammad Nasb (MSc)^#12^, Sayed Zulfiqar Ali Shah (M.S) ^# 1^,Liangjiang Huang^1,3^, Qian Li^1,3^ , Hong Chen (PhD)*^1,3^

^1^ Department of Rehabilitation Medicine, Tongji Hospital, Tongji Medical College, Huazhong University of Science and Technology, Wuhan 430030, People’s Republic of China.

^2^ Department of Physical Therapy, Health science faculty, Albaath University, Homs, Syria.

^3^ WHO Collaborating Centre for Training and Research in Rehabilitation, Tongji Hospital, Tongji Medical College, Huazhong University of Science and Technology, Wuhan 430030, People’s Republic of China.

# the two authors equally contributed to the manuscript

***Corresponding Author:** Dr. Hong Chen

**Email address:** 1432133443@qq.com, chenhong1129@hust.edu.cn

**Address:** Department of Rehabilitation Medicine, Tongji Hospital, Tongji Medical College, Huazhong University of Science and Technology, Wuhan 430030, People’s Republic of China.

**Abstract**

**Background**

Arising pathogens are a worldwide challenge for [healthful living](https://www.thesaurus.com/browse/healthful%20living). Severe acute respiratory syndrome coronavirus 2 (COVID-19) is an enveloped RNA virus that distributed dramatically among humans. COVID-19 mainly attacks the respiratory system leading to severe acute respiratory syndrome and pneumonia. Some Rehabilitation modalities such as short waves diathermy could be of help in supporting the medical treatment and avoiding complications of COVID-19 pneumonia.

**Methods/design:** This is a single center, evaluator blinded, parallel design randomised, controlled clinical trial study protocol based on SPIRIT guidelines. A Randomization plan will be created online on [www.randomization.com](http://www.randomization.com) by a statistician who will not be part of the study. Small blocks of various sizes will be used. A total of 410 patients will be randomised in 1:1 ratio to two groups: experimental group (n=205) and control group (n=205) by permuted blocks method. The primary outcome measures will be time to positive-to-negative conversion of COVID-19 nucleic acid test in pharyngeal swab, in days assessed at 7^th^, 14^th^ ,21^st^ and 28^th^ day. The secondary outcome measures include nucleic acid test rate and recovery from symptoms, Vital signs assessment, Computed Tomography, Complete blood count, Creatine kinase, Lactate dehydrogenase, Inflammatory cytokines, Inflammatory Markers, Immune Markers and SIRS scale scores. Blinded evaluation will be at baseline (the day of starting ultra-short-wave diathermy) and after 28 days following the interventions.

**Objective:** To evaluate the therapeutic effects of ultra-short-wave diathermy (SWD) on COVID-19 pneumonia.

**Discussion**: This clinical trial will be the first randomised controlled clinical trial to comprehensively examine the efficacy of ultra-short-wave diathermy on COVID-19 pneumonia patients. The results may provide quality evidence of the role of ultra-short-wave diathermy in improving outcomes and avoiding complications of this viral infection.

**Clinical Study registration:** This randomised controlled trial had been prospectively registered with the Chinese Clinical Trials (ChiCTR2000029972) at 17, February ,2020. URL: http://www.chictr.org.cn/historyversionpuben.aspx?regno=ChiCTR2000029972

**Keywords**: COVID-19, coronavirus, Ultra short-wave diathermy, Pneumonia

**Background**

Betcoronaviruses are single strand RNA viruses hosted in variety of animals, especially mammals. The Severe acute respiratory syndrome coronavirus 2 (COVID-19), emerged as a fifth novel coronavirus in Wuhan, Hua nan sea food market [1].

This novel strain causes mild to severe pneumonia (COVID-19), initially reported in Wuhan, Central China [2-4]. The COVID-19 rapidly spread in China causing infection in 74283 people on the Mainland China, with 2009 (2.7%) deaths so far (February 19,2020) [5, 6].

As it is clear that COVID-19 can transmit from human to human [7], it was predicted that the extensive migration before Chinese New Year starting spiked the spread of virus massively to other parts of China [8]. It was suggested that COVID-19 transmitted to humans from its primary host bat through an intermediate host (non bat animals), linking to the medical histories of a cluster of infected patients suspected to be infected in Hua Nan market where wild animals are sold [9]. This strongly suggests the infection of human from the wild animals in the market and later infected people spread it to others. But the conclusion about original source, intermediate host and the originating point is still debatable because some case were found to have no relation with Hua Nan market [5, 7].

The most common initial clinical symptoms of Wuhan COVID-19 victims reported by a recent study were fever, cough, body ache or fatigue, while the less common symptoms were hemoptysis, diarrhea and headache. Some patients later developed shortness of breath, lymphopenia, while all recruited patients had pneumonia later [5]. Clinical features of 138 COVID-19 patients reported were shock, cardiac injury, acute kidney injury, acute respiratory distress syndrome and arrhythmias in many cases [6].

Till date no specific treatment exist to manage coronavirus victims [10], the present treatment is only supportive to treat the symptoms of the affected patients. The current treatment protocol includes antiviral therapy, corticosteroid therapy, respiratory support, kidney replacement therapy, Oxygen inhalation, invasive mechanical ventilation, non-invasive ventilation, extracorporeal membrane oxygenation [6].

Evidence supports the utilization of certain rehabilitation techniques like chest physiotherapy and shortwave diathermy (SWD) in the management of pneumonia and severe acute respiratory distress syndrome [11-13]. As it is known, rising temperature decline the activity and viability of viruses, SWD is among the common and essential physiotherapy modalities [13, 14]. SWD can produce an efficient deep heating in the treated area within the physiologic limits. SWD could roughly raises the muscle temperature around 15**°**C [15] in vitro / vivo studies had investigated the safety and efficacy of using SWD as a bactericidal or virucidal [16, 17]. The main principle of SWD is a non-specific action to rise the micro-organisms temperature above its thermally death point [15].

SWD also has the potential to penetrate directly into the lungs, which can promote the secretion of local anti-inflammatory factors, accelerate local blood circulation, and promote the drug to enter the infected site to exert better curative effects [13]. Moreover, SWD can significantly increase the phagocytic activity and adherence capacity of leukocytes, which increase exponentially with increasing temperature up to 40 ° C. This supports the use of short waves in early pneumonia to stimulate and enhance the body's natural defenses against microorganisms.

In 2003 SWD treatment had been hired during the SARS outbreak through combining shortwave diathermy with conventional therapy among 38 cases. The results showed that the use of short-wave as an adjuvant therapy for drug therapy has obvious effects. Utilizing short waves can speed up patient recovery and shorten hospital stays [18, 19]. Thus, according to earlier studies, using SWD could be of benefit in such viral infectious [16]. The purpose of this study is to investigate the effectiveness of Ultra shortwave diathermy in managing COVID-19(pneumonia). If shown to be effective, this intervention could be utilized for many patients, to ease up the COVID-19 associated symptoms, reduce complications and mortality, enhance recovery and decrease healthcare costs.

**Methods**

**Study design**

This is single center, evaluator blinded, parallel design randomised, controlled clinical trial study protocol, designed following the SPIRIT 2013 statement. Patient recruitment, the randomization plan, and the study events are visually described in Figure no.1.The current study was approved by the Tongji Hospital ethics committee and prospectively registered with the Chinese Clinical Trials Registry (Registry no. ChiCTR2000029972). Due to treatment nature, treatment providers and patients couldn’t be blinded in this study. However, patient evaluators and data collectors will be blinded to the intervention and group allocation. This trial will be carried out in accordance with the requirements of the "Good Clinical Practice for Drug Control (GCP)", the Helsinki Declaration and relevant laws and regulations. The intervention will be carried out in accordance with GCP, Standard Operating Procedures for clinical trials (SOP), and quality control requirements. The participants will be randomly divided into two groups: experimental group (n=205), control group (n=205). The experimental group will receive Ultra short-wave diathermy along with standard medical treatment for COVID-19 and the control group will be given only standard medical treatment for COVID-19.

**Figure no.1 Study Flow Chart**


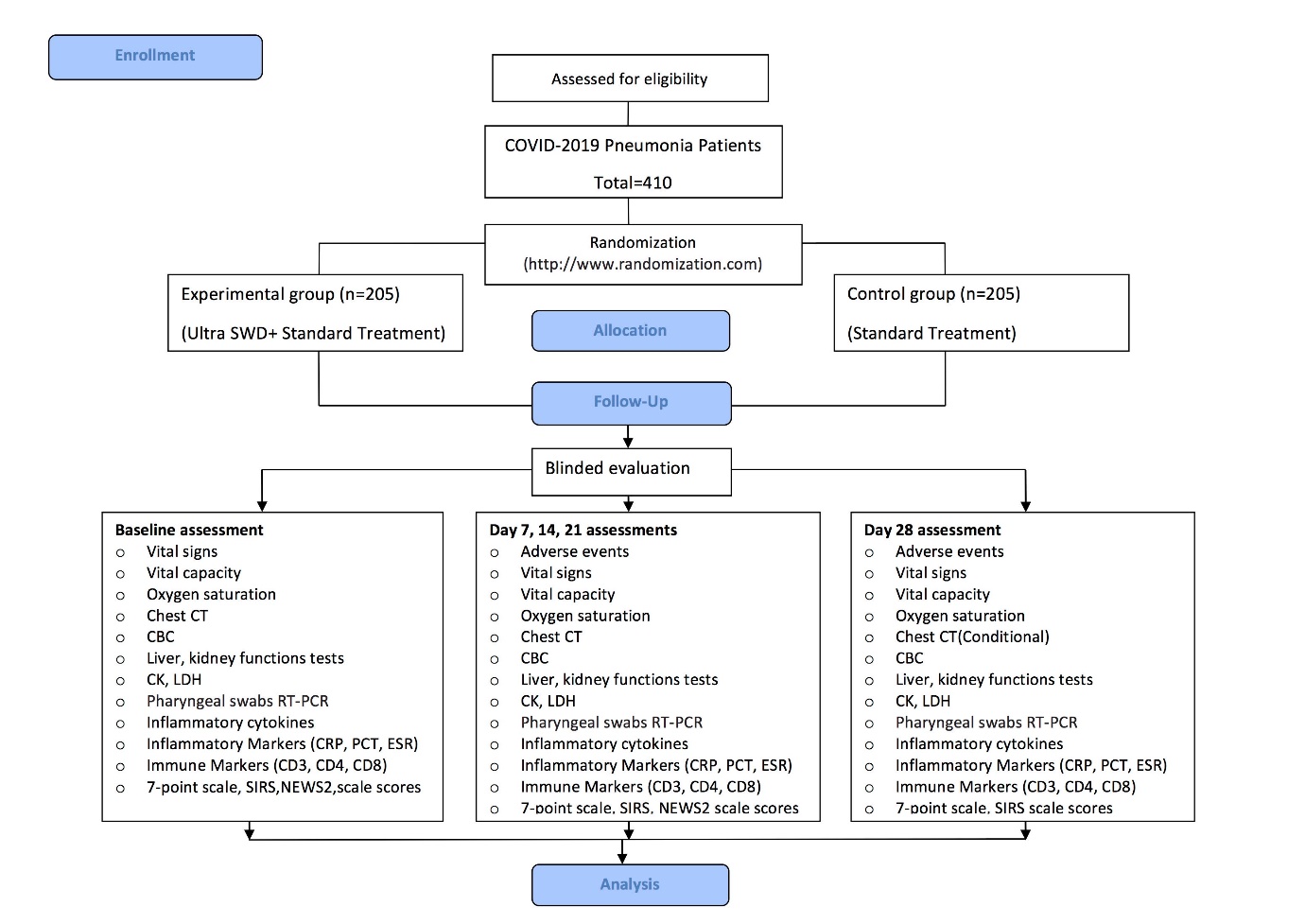
Figure no.1 CONSORT Flow Diagram demonstrating patient flow through different events throughout the trial, Computed Tomography(CT), Complete blood count(CBC), Creatine kinase (CK) ,lactate dehydrogenase (LDH), Erythrocyte sedimentation rate ( ESR), C-reactive protein ( CRP ),  Procalcitonin ( PCT ), Cluster of Differentiation(CD), National Early Warning Score 2 (NEWS2), Systemic Inflammatory Response (SISR)

**Study population recruitment**

Patients of all genders with age range from 18 to 65 diagnosed positive for COVID-19 will be recruited from the Tongji University Hospital (Huazhong University of Science and Technology, Wuhan, China). We selected the age range 18-65 because, firstly most of the COVID-19 cases reported in Wuhan were in the given age range, and secondly patients above 65 were having a higher rate of intensive care unit (ICU) admission.

Participants will undergo preliminary screenings according to the eligibility criteria. Eligible participants will be invited to take the baseline assessment to confirm that they meet the inclusion criteria and provide written informed consent to participate. After this step, participants will be randomly divided into two groups: experimental group and a control group.

**Inclusion criteria:**

(1) Age 18-65 years.

(2) Coronavirus nucleic acid test is positive.

(3) Lung CT showed multiple patchy ground glass shadows or other typical manifestations of both lungs.

**2. Exclusion criteria:**

(1) Positive tests for other pathogens such as Tuberculosis, Mycoplasma.

(2) Patients with respiratory failure or requiring mechanical ventilation.

(3) People with metal implants or pacemaker.

(4) Those with shock.

(5) Those that have bleeding tendency or active bleeding in the lungs.

(6) Patients with multiple organ failure who need ICU monitoring and treatment.

(7) Cancer patients and those with severe underlying diseases.

(8) Pregnant or lactating women.

(9) Patients with severe cognitive impairment who cannot follow the instructions to complete the treatment.

(10) Those without signed informed consent.

(11) Those with other contraindications to short wave.

**Sample size**

The sample size for this randomised controlled trial was calculated by GPower 3.1 by using A-priori sample size calculation. Based on the values for our main variable, time to positive-to-negative conversion of COVI-19 nucleic acid test in pharyngeal swab, from another recently published study, using α=0.05, 80% power, and an effect size of 0.28, an enrolment of 390 participants would be enough to detect a statistically significant difference in time to positive-to-negative conversion of COVI-19 nucleic acid test between the two groups. Moreover 20 patients will be added to total sample to manage the expected 5% dropouts, making the total sample size 410.

**Randomization**

A Randomization plan will be created online on [www.randomization.com](http://www.randomization.com) by a statistician who will not be part of the study. A total of 410 participants will be randomised into a single treatment using randomly permuted blocks method. Small blocks of various sizes will be used so that randomization could less likely be guessed. Patients will randomly be distributed into one of the two groups in a ratio of 1:1. The randomization will be implemented with opaque envelopes.

**Blinding**This study is an evaluator blinded study. The blinding of both participants and healthcare workers is not possible in such type of intervention. Only the patient and the study physician know the randomization outcome. Other team members, patient evaluators and data collectors are blinded. After finishing the study, statistician will be present un-blinded data.

**Treatment Protocol**

**1. Groups and intervention procedures:**

After signing the informed consent, all patients will be randomly divided into the following two groups: the experimental group will be given the nationally recommended standard drug treatment + ultra-short-wave diathermy treatment; the control group will be given only the nationally recommended standard drug treatment. Each group is planned to include 205 participants.

**Short wave treatment dose:** Ultra-short-wave therapy treatment will be performed through application of ultra-short-wave therapy machine electrodes on the anterior and posterior parts of the trunk for 10 minutes, twice a day for 12 consecutive days.

**Outcome measures**

The primary outcome measures will be time to positive-to-negative conversion of COVID-19 nucleic acid in pharyngeal swab, in days. The secondary outcome measures include nucleic acid test rate and recovery from symptoms , vital signs assessment, treatment adverse effects, Vital capacity, Oxygen saturation, Computed Tomography(CT), Complete blood count(CBC), Creatine kinase (CK) ,Lactate dehydrogenase (LDH), Pharyngeal swabs RT-PCR, Inflammatory cytokines, Inflammatory Markers (Erythrocyte sedimentation rate ( ESR), C-reactive protein ( CRP ),  Procalcitonin ( PCT )), Immune Markers (CD3, CD4, CD8) and SIRS scale scores measured at baseline, after 7 days, 14 days, 21 days, and 28 days of treatment.

**Data collection tools**

Separate data collection forms are developed for trial data collection as: Medical history forms for taking relevant medical history, Case Report Form (CRF) to collect treatment related data, adverse events forms to collect data regarding any adverse event happening during the trial. For recording improvement in clinical symptoms 7-category ordinal, SIRS and NEWS2 scales will be used.

**The 7-category ordinal**

The 7-category or 7-point ordinal scale is consisting of seven separate categories. The categories range from 1-7(1,discharged from the hospital and can resume normal functions,7, death) given as: category 7 corresponds to death; category 6 patients need ICU hospitalization, require ECMO and/or invasive mechanical ventilation; category 5 patients need ICU hospitalization but do not require ECMO and/or invasive mechanical ventilation; category 4 patients are non-ICU hospitalized patients, requiring supplemental oxygen; category 3 patients are also non-ICU hospitalized patients but they do not require supplemental oxygen; category 2 patients are not hospitalized, but unable to resume normal activities; category 1, not hospitalized with resumption of normal activities [20].

| **Description** | **0 points** | **1 points** | **2 points** | **3 points** | **4 points** | **score** |
| --- | --- | --- | --- | --- | --- | --- |
| Heart beat | 60-100 | 55-59;  100-119 | 50-54;  120-140 | 41-49;  141-160 | <40;  >160 |  |
| MAP(mmHg) | 70-100 | 60-69;  101-110 | 50-59;  111-130 | 40-49;  131-159 | <40;  >160 |  |
| Respiratory rate / min | 12-20 | 9-12;  20-25 | 5-8;  26-35 | <5;  36-45 | 0;  >46 |  |
| SpO_2_（%） | >92 | 85-91 | 75-84 | 60-74 | <60 |  |
| body temperature（℃） | 36.0-37.5 | 35-35.9;  37.5-38.5 | 34-34.9;  38.6-39.5 | 33-33.9;  39.6-40 | <33;  >40 |  |
| WBC(*10^9^/L) | 4.0-10.0 | 3.0-3.9;  10.1-14.9 | 2.0-2.9;  15-20.0 | 1.0-2.0;  20.1-30.0 | <1.0;  >30.1 |  |
| GLU(mmol/L) | 3.5-5.5 | 5.7-8.6 | 8.7-13.5 | 13.6-23 | >23 |  |
| Level of consciousness | Aware/awake | Lethargy or irritability | Shallow coma | coma | brain death |  |
| **Total score :** | | | | | |  |

**SIRS**

Systemic Inflammatory Response (SIRS) Scale is used for evaluation of clinical improvement based on Heart rate, Mean arterial pressure(MAP mmHg), Respiratory rate / min, Blood oxygen saturation (SpO_2_ %）, body temperature（℃）, White blood cells (WBC *10^9^/L), Blood glucose (mmol/L) and Level of consciousness (Aware/awake, Lethargy or irritability, Shallow coma, coma, brain death). All these parameters except “Level of consciousness” are assigned a score from 0 to 4 based on the actual values recorded from patient corresponding to the range of values in Table no.1. For level of consciousness: Aware/awake (0), Lethargy or irritability (1), Shallow coma (2), coma (3), brain death (4).

**Table no.1: Systemic Inflammatory Response (SISR) Scale**

**NEWS2**

National Early Warning Score 2 (NEWS2) is commonly used for acute illness assessment based on aggregate scoring system from six physiological parameters which form the basis of scoring (respiration rate, oxygen saturation, systolic blood pressure, pulse rate, level of consciousness or new confusion, temperature). The magnitude of score allocated to every parameter shows its deviation from the normal. The score is summed and 2 points added for people requiring supplemental oxygen. The score is further categorized as: LOW score (an aggregate of 1–4), MEDIUM score (an aggregate of 5 or 6) and a HIGH score (an aggregate of 7 or more) [21].

**Clinical Observation**

**Before treatment (day -1 ~ 1):**

● Evaluation and recording of demographic data, vital signs (pulse, respiration, blood pressure, body temperature), blood oxygen saturation, and vital capacity.

● Medical history: including current medical history, past medical history and drug allergy history.

● Laboratory tests: coronavirus nucleic acid test, Complete blood count (CBC), Creatine kinase (CK), Lactate dehydrogenase (LDH), Pharyngeal swabs RT-PCR, Inflammatory cytokines, Inflammatory Markers (Erythrocyte sedimentation rate (ESR), C-reactive protein (CRP), Procalcitonin (PCT), Immune Markers (CD3, CD4, CD8).

● Radiologic examination: Chest CT

● Other tests: ECG

● Combined medications.

● Complete the 7-category ordinal scale and SIRS scale

**Treatment period (days 7, 14, 21)**

● Evaluate and record vital signs (pulse, respiration, blood pressure, body temperature), blood oxygen saturation and vital capacity

● Laboratory tests: coronavirus nucleic acid test, Complete blood count (CBC), Creatine kinase (CK), Lactate dehydrogenase (LDH), Pharyngeal swabs RT-PCR, Inflammatory cytokines, Inflammatory Markers (Erythrocyte sedimentation rate (ESR), C-reactive protein (CRP), Procalcitonin (PCT), Immune Markers (CD3, CD4, CD8).

● Radiologic examination: Chest CT

● Other tests: ECG

● Complete symptoms evaluation: SIRS scale score, the 7-category ordinal scale, and The National Early Warning Score 2 (NEWS2)

**Follow-up period (day 28)**

● Evaluate and record, vital signs (pulse, respiration, blood pressure, body temperature), blood oxygen saturation, vital capacity.

● Laboratory tests: coronavirus nucleic acid test, Complete blood count (CBC), Creatine kinase (CK), Lactate dehydrogenase(LDH), Pharyngeal swabs RT-PCR, Inflammatory cytokines, Inflammatory Markers (Erythrocyte sedimentation rate ( ESR), C-reactive protein ( CRP ), Procalcitonin ( PCT ), Immune Markers (CD3, CD4, CD8).

● Radiologic examination: CT scan of the lungs is available to those who have the condition

● Other tests: ECG

● Complete symptoms evaluation: SIRS scale score and 7-category ordinal scale.

**Criteria for termination of clinical trial**

During the intervention, if the participants have the following circumstances, the intervention must be suspended, specifically:

(1) For those with intolerable adverse events / serious adverse events, if the investigator judges that the risk to the patient on continuing to participate in the trial is greater than the benefit, the trial must be suspended and appropriate treatment measures should be taken in time.

(2) Patients who have exacerbated conditions after treatment, but should be included in the efficacy and safety analysis of the protocol set.

(3) Patients who changed mind about participation in the trial due to any reason or no more willing to participate.

The investigator or sponsor has the right to terminate the study in advance, but the patient must be consulted on the relevant matters of enrollment, report to the ethics committee, and explain the reason. The trial can be terminated early after obtaining consent. For subjects who are excluded or suspended early, the date and reason for the suspension shall be recorded in the corresponding table. Participants who are excluded or terminated early will not be replaced by new subjects, and once they withdraw from the trial, they will not be able to re-enter the study. All adverse events and abnormal laboratory indicators will be followed up to pre-treatment status or to no clinical significance.

Once the trial is terminated, all research materials (including completed, partially completed, and blank case report forms), research equipment, and research drugs shall be returned to the sponsor.

**Preservation of case report form**

The primary researcher will keep all the detailed original documents of the subjects, and record in the case report forms the treatment progress, medication, laboratory inspection data, safety data, and efficacy evaluation timely and clearly. Case report forms, original documents, medical records, etc. should be clear, detailed, and easily identifiable by personnel participating in this clinical trial. The lead investigator will sign the confirmation and completion pages of the case report forms to verify the accuracy and completeness of all data. The case report forms and the original files will only be modified by the investigator. Any modification to the case report forms and the original file must not obliterate the original data. The correct modification method is to draw a single line on the original data, then write the modified data next to the original data, and sign the date and the initials of the person who modified it.

The trail data shall be retained for 10 years after the end of the trial. However, if required by current regulations or an agreement with the sponsor, this information should be kept for a longer period of time.

**Adverse event records, reports and treatments**

**1. Definition of adverse events**

An adverse event is any undesired medical event that occurs during or after treatment to a patient in a clinical study. All adverse events do not necessarily have a causal relationship with the treatment. Since short wave diathermy is routinely practiced for pneumonia treatment in our rehabilitation department, no large scale adverse events have been found in routine treatments of pneumonia.

**2. Reporting requirements**

Researchers will evaluate each adverse event to determine whether it meets the criteria for serious adverse events. If a serious adverse event occurs, it will be reported quickly in accordance with national regulations. All adverse events will be recorded on the adverse event page of the case report form. It should be noted that the report form used to collect information on serious adverse events is different from the case report form that just has a page “adverse event record page” for recording adverse events. The serious adverse events report form is shown in the appendix. In addition, because the data collected are same, the two forms must be filled accordingly. For example, the same adverse event terms should be used in both forms. Regardless of whether filling out a medical report form or an adverse event report form, same adverse event terms and accurate medical terms should be used in reporting adverse events.

**3. Reporting requirements for serious adverse events**

If a serious adverse event occurs during the trial, regardless of its relation to the treatment method, relevant treatment measures will be taken immediately and reported to the main investigator (Chen Hong), project leader and ethics committee (Ethics Committee of Tongji Hospital of Huazhong University of Science and Technology) within 24 hours.

**Statistical methods**

Data collection will be performed after the patient is discharged from the Hospital, and completed by the study data collector separately; a member of the study team, blinded to the intervention groups. Adjudication of any conflicting data points will be identified by the study statistician and reviewed in tandem by the data collector to settle any discrepancies. The data will be available upon reasonable request from the corresponding author.

**Data Analysis Plan**

The principles of intent-to-treat analysis will be followed to include all participants randomised for the study. Data normality will be assessed using the Kolmogorov-Smirnov (K-S) and Shapiro-Wilk (S-W) tests. The missing data will be managed by multiple imputations. Descriptive statistics: mean, median, maximum, minimum, confidence interval, and standard deviation will be calculated for continuous data, and frequency distributions will be calculated for categorical data. Prevalence of negative and positive tests will be calculated at specific time points. Independent t test will be used to compare the means after intervention between the two groups in case of normal distribution of data. If the data was not normally distributed Mann Whitney test will be used. Chi-square test will be used for comparison of categorical data between the two study groups.

Linear regression analysis will be used to evaluate any relationship between age, gender and study groups and the primary (time to positive-to-negative conversion of COVID-19 nucleic acid test) and some secondary variables. Adjusted and unadjusted analysis will be performed. Compliance analysis: Statistics whether the patients were treated with short wave on time. According to the requirements of the adverse reaction correlation, we will list the adverse events and adverse reactions of the two groups (including the number of various adverse events, laboratory testing indicators before and after the treatment "normal to abnormal" or” "Aggravated abnormally"), the number of cases and the rate of change, list the reasons and explain. Chi-square test will be used for statistical analysis of adverse reactions. Within the group and between the groups analysis will be performed using 95% confidence internal and a p value of 0.05. Analysis of influencing factors: If there are significant differences between the two groups before treatment, such as age, gender, disease type, and condition, or there are related factors (such as combined medication) that significantly affect the effects of treatment during the treatment, the comparison of the efficacy, these factors will be considered as covariates, and analysis of covariance or logistic regression will be is used. The analyzed data will mainly be expressed in the form of self-explanatory tables with statements, table notes. The results of repeated measurement data will be expressed in both tables and statistical charts to increase readability.

**Statistics software**

Simultaneous analysis will be performed using SPSS version 26 and GraphPad prism 8.

**Treatment quality**

The quality control of this intervention was jointly controlled by the person in charge of this study and the Ethics Committee of Tongji Hospital of Tongji Medical College of Huazhong University of Science and Technology. The research work will be performed in accordance with GCP, SOP and quality control requirements. We have developed a clinical research plan and conducted GCP training for relevant researchers participating in this trial.

1) The scheme will be submitted to the Ethics Committee of Tongji Hospital affiliated to Tongji Medical College of Huazhong University of Science and Technology for approval before the trial begins.

2) According to GCP guidelines, necessary steps should be taken during the design and implementation of the study to ensure that the data collected is accurate, consistent, complete and credible.

3) Participating researchers will conduct the intervention in strict compliance with China's GCP standards, and in accordance with the clinical trial standard operating procedures (SOP), truthfully, carefully, and carefully collect and record the contents of the case report form and verify them to ensure the data's accuracy and reliability.

4) The researcher will fill in the information required by the scheme into the case report form (CRF), and the inspector will verify whether the completion is complete and accurate, and instructs the staff of the research center to make necessary corrections and additions.

5) Various instruments, equipment, reagents, standards used in these clinical trials will be inspected following strict quality standards to ensure that they work under normal conditions.

6) The statistical problems in the treatment plan are sent to a statistical expert for review and control

7) During the trial, the investigator will monitor the research process, informed consent, and correctness and completeness of the data in the case report form (CRF).

**Discussion**

Patients diagnosed positive for COVID-2019 will be recruited from Tongji Hospital to participate in this study in rehabilitation department of Tongji Hospital. We will attempt to standardize all treatments and outcomes measurements by performing it at the same time of the day under the instructions and supervision of a trained doctor. The purpose of this study is to investigate the effectiveness of Ultra SWD in managing COVID-2019 patients. We are also aiming to encourage other healthcare professionals to include Ultra SWD in their daily practice of COVID-2019 patients management to treat their clients effectively.

**Conclusion**

To the best of our knowledge, the present study design will be the first randomised clinical trial evaluating the efficacy of Ultra SWD for treatment of COVID-2019(Pneumonia) patients. Our trial design and sample size adopted ensures the production of reliable and generalizable results.

**Abbreviations**

Short Wave Diathermy (SWD)

Good Clinical Practice for Drug Control (GCP)

Intensive Care Unit (ICU)

Randomised Controlled Trial (RCT)

Standard Operating Procedures (SOP)

Severe Acute Respiratory Syndrome Coronavirus 2 (COVID-19 )

Coronavirus Disease 2019 (COVID-19)

Standard Protocol Items: Recommendations for Interventional Trials (SPIRIT)

Computed Tomography (CT)

Complete Blood Count (CBC)

Creatine Kinase (CK)

Lactate Dehydrogenase(LDH)

Reverse Transcription Polymerase Chain (RT-PCR)

Erythrocyte Sedimentation Rate (ESR)

C-Reactive Protein (CRP)

Procalcitonin (PCT)

Cluster of differentiation (CD)

Electrocardiogram (ECG)

Systemic Inflammatory Response Syndrome (SIRS)

Case Report Form (CRF)

**Declarations**

**Trial status**

Protocol version 1 was approved on 02/12/2020.This trial was registered online on 02/17/2020 The recruitment for this trial completed on 30/03/2020. The trial is expected to complete in July 2020.

**Ethics approval and consent to participate**

This research follows the Declaration of Helsinki and has been approved by the Ethics Committee of Tongji Hospital. Also, an informed consent will be obtained from all study participants.

**Consent for publication**

Written consent will be received from all participants to publicly share the results of the study.

**Availability of data and materials**

The datasets produced during this study are not publicly available as the study status is currently ongoing. The data set will be made available upon reasonable request to corresponding author after completion of the study.

**Competing interests:** The authors declare that they have no competing interests.

**Funding:** The National Natural Science Foundation, an external peer-review took place during the funding process. The funder had no role in the design and conduct of the study; collection, management, analysis, and interpretation of the data; preparation, review, or approval of the manuscript; and decision to submit the manuscript for publication.

**Authors’ contributions**

Authors MN and SZAS equally contributed to designing, writing, formatting of this study. Author LH and LQ contributed in drafting of the final manuscript and will assist in data collection for the trial. Author HC contributed to conception, designing and supervision of the study. All authors have read and approved the manuscript.

**Acknowledgements**

Our thanks to all the team at Tongji Hospital and research center where the trial will take place.

**References**

1. Cui J, Li F, Shi Z-L. Origin and evolution of pathogenic coronaviruses. Nature reviews Microbiology 2019, 17(3):181-192.

2. Zhou P, Yang X-L, Wang X-G, Hu B, Zhang L, Zhang W, Si H-R, Zhu Y, Li B, Huang C-L. A pneumonia outbreak associated with a new coronavirus of probable bat origin. Nature 2020:1-4.

3. Zhu N, Zhang D, Wang W, Li X, Yang B, Song J, Zhao X, Huang B, Shi W, Lu R. A novel coronavirus from patients with pneumonia in China, 2019. New England Journal of Medicine 2020.

4. Chen N, Zhou M, Dong X, Qu J, Gong F, Han Y, Qiu Y, Wang J, Liu Y, Wei Y. Epidemiological and clinical characteristics of 99 cases of 2019 novel coronavirus pneumonia in Wuhan, China: a descriptive study. The Lancet 2020.

5. Huang C, Wang Y, Li X, Ren L, Zhao J, Hu Y, Zhang L, Fan G, Xu J, Gu X. Clinical features of patients infected with 2019 novel coronavirus in Wuhan, China. The Lancet 2020.

6. Wang D, Hu B, Hu C, Zhu F, Liu X, Zhang J, Wang B, Xiang H, Cheng Z, Xiong Y et al. Clinical Characteristics of 138 Hospitalized Patients With 2019 Novel Coronavirus–Infected Pneumonia in Wuhan, China. JAMA 2020.

7. Li Q, Guan X, Wu P, Wang X, Zhou L, Tong Y, Ren R, Leung KS, Lau EH, Wong JY. Early transmission dynamics in Wuhan, China, of novel coronavirus–infected pneumonia. New England Journal of Medicine 2020.

8. Wu JT, Leung K, Leung GM. Nowcasting and forecasting the potential domestic and international spread of the 2019-nCoV outbreak originating in Wuhan, China: a modelling study. The Lancet 2020.

9. Lam TT-Y, Shum MH-H, Zhu H-C, Tong Y-G, Ni X-B, Liao Y-S, Wei W, Cheung WY-M, Li W-J, Li L-F. Identification of 2019-nCoV related coronaviruses in Malayan pangolins in southern China. bioRxiv 2020.

10. de Wit E, van Doremalen N, Falzarano D, Munster VJ: SARS and MERS. recent insights into emerging coronaviruses. Nature Reviews Microbiology 2016, 14(8):523.

11. Blackwood S. Shortwave diathermy (SWD) in the treatment of unresolved pneumonia. South African Journal of Physiotherapy 1980, 36(3):72-73.

12. Kim SJ, Lee JH, Han B, Lam J, Bukowy E, Rao A, Vulcano J, Andreeva A, Bertelson H, Shin HP. Effects of hospital-based physical therapy on hospital discharge outcomes among hospitalized older adults with community-acquired pneumonia and declining physical function. Aging and disease 2015, 6(3):174.

13. Zhang L feng, Zheng G xin, Liu G lin. Ultra short wave therapy in the treatment of severe acute respiratory syndrome (SARS) .Chinese Journal of Physical Medicine and Health Complex, 2003, 25 (6): 332-334. http://www.cnki.com.cn/Article/CJFDTotal-ZHLY200306007.htm

14. Hou A, Yang Z, Gao C, Jiang H, Huang J. The effect of short wave diathermy on dendritic cells in hepatitis B virus transgenic mice. Chinese Journal of Physical Medicine and Rehabilitation 2003.

15. Shields N, Gormley J, O'Hare N. Short-wave diathermy in Irish physiotherapy departments. In.: MA Healthcare London; 2001.

16. Shields N, Gormley J, O'Hare N. Short-wave diathermy: a review of existing clinical trials. Physical therapy reviews 2001, 6:101-118.

17. Pope GD, Mockett SP, Wright JP. A survey of electrotherapeutic modalities: ownership and use in the NHS in England. Physiotherapy 1995, 81:82-91.

18. Mortimer B, Beard G. Tissue heating by short wave diathermy. Physical Therapy 1935, 15:229.

19. Coulter JS. Medical diathermy. Journal of the American Medical Association 1936, 106:209-214.

20. Wang Y, Fan G, Horby P, Hayden F, Li Q, Wu Q, Zou X, Li H, Zhan Q, Wang C. Comparative outcomes of adults hospitalized with seasonal influenza A or B Virus infection: application of the 7-Category Ordinal Scale. In: Open forum infectious diseases: 2019: Oxford University Press US; 2019: ofz053.

21. Williams B, Alberti G, Ball C, Ball D, Binks R, Durham L. Royal College of Physicians, National Early Warning Score (NEWS), Standardising the assessment of acute-illness severity in the NHS, London. 2012.
